# Incidence, prevalence and mortality rates of malaria in Ethiopia from 1990 to 2015: analysis of the global burden of diseases 2015

**DOI:** 10.1186/s12936-017-1919-4

**Published:** 2017-07-04

**Authors:** Amare Deribew, Tariku Dejene, Biruck Kebede, Gizachew Assefa Tessema, Yohannes Adama Melaku, Awoke Misganaw, Teshome Gebre, Asrat Hailu, Sibhatu Biadgilign, Alemayehu Amberbir, Biruck Desalegn Yirsaw, Amanuel Alemu Abajobir, Oumer Shafi, Semaw F. Abera, Nebiyu Negussu, Belete Mengistu, Azmeraw T. Amare, Abate Mulugeta, Birhan Mengistu, Zerihun Tadesse, Mesfin Sileshi, Elizabeth Cromwell, Scott D. Glenn, Kebede Deribe, Jeffrey D. Stanaway

**Affiliations:** 1 St. Paul Millennium Medical College, Addis Ababa, Ethiopia; 2 Dilla University, Dilla, Ethiopia; 3 Nutrition International (former Micronutrient Initiative), Addis Ababa, Ethiopia; 4 Center for Population Studies, Addis Ababa University, Addis Ababa, Ethiopia; 5 Federal Ministry of Health, Addis Ababa, Ethiopia; 6 Department Reproductive Health, Institute of Public Health, University of Gondar, Gondar, Ethiopia; 7 School of Public Health, The University of Adelaide, Adelaide, Australia; 8 School of Medicine, The University of Adelaide, Adelaide, SA Australia; 9 School of Public Health, Mekelle University, Mekelle, Ethiopia; 10 Institute of Health Metrics and Evaluation, University of Washington, Seattle, USA; 11 International Trachoma Initiative, The Task Force for Global Health, Addis Ababa, Ethiopia; 12 School of Medicine, Addis Ababa University, Addis Ababa, Ethiopia; 13 World Health Organization, Kampala, Uganda; 14 Dignitas International, Medical and Research Department, Zomba, Malawi; 15 University of South Australia, Adelaide, Australia; 16 School of Public Health, The University of Queensland, St Lucia, QLD Australia; 17 Debremarkos University, Debremarkos, Ethiopia; 18 Rollins Schools of Public Health, Emory University, Atlanta, USA; 19 Institute of Biological Chemistry and Nutrition, Hohenheim University, Stuttgart, Germany; 20 College of Medicine and Health Sciences, Bahir Dar University, Bahir Dar, Ethiopia; 21 World Health Organization, Addis Ababa, Ethiopia; 22 The Carter Centre, Addis Ababa, Ethiopia; 23 Wellcome Trust Brighton and Sussex Centre for Global Health Research, Brighton and Sussex Medical School, Falmer, Brighton, UK; 24 School of Public Health, Addis Ababa University, Addis Ababa, Ethiopia

## Abstract

**Background:**

In Ethiopia there is no complete registration system to measure disease burden and risk factors accurately. In this study, the 2015 global burden of diseases, injuries and risk factors (GBD) data were used to analyse the incidence, prevalence and mortality rates of malaria in Ethiopia over the last 25 years.

**Methods:**

GBD 2015 used verbal autopsy surveys, reports, and published scientific articles to estimate the burden of malaria in Ethiopia. Age and gender-specific causes of death for malaria were estimated using cause of death ensemble modelling.

**Results:**

The number of new cases of malaria declined from 2.8 million [95% uncertainty interval (UI) 1.4–4.5 million] in 1990 to 621,345 (95% UI 462,230–797,442) in 2015. Malaria caused an estimated 30,323 deaths (95% UI 11,533.3–61,215.3) in 1990 and 1561 deaths (95% UI 752.8–2660.5) in 2015, a 94.8% reduction over the 25 years. Age-standardized mortality rate of malaria has declined by 96.5% between 1990 and 2015 with an annual rate of change of 13.4%. Age-standardized malaria incidence rate among all ages and gender declined by 88.7% between 1990 and 2015. The number of disability-adjusted life years lost (DALY) due to malaria decreased from 2.2 million (95% UI 0.76–4.7 million) in 1990 to 0.18 million (95% UI 0.12–0.26 million) in 2015, with a total reduction 91.7%. Similarly, age-standardized DALY rate declined by 94.8% during the same period.

**Conclusions:**

Ethiopia has achieved a 50% reduction target of malaria of the millennium development goals. The country should strengthen its malaria control and treatment strategies to achieve the sustainable development goals.

## Background

Ethiopia has registered remarkable progress in reducing the burden of malaria and other major communicable diseases over the last two decades [1, 2]. Over the last decade, the burden of malaria has declined significantly, which could be the result of improved coverage of high impact interventions, such as prompt treatment of cases using artemisinin-based combination therapy (ACT), prevention and control of malaria among pregnant women using intermittent preventive therapy (IPT), use vector control methods including insecticide-treated bed nets (ITNs), and indoor residual spray (IRS) [3–5]. Malaria deaths and admissions in children age under-5 fell by 81 and 73%, respectively, after the scale-up of ITNs, IRS and ACT interventions between 2006 and 2011 [4]. However, malaria remains a major health problem for Ethiopia where only 25% of the population live in areas that are free from malaria [6, 7]. It is still among the ten top leading causes of morbidity and mortality in children under-5 years [8].

The World Health Organization (WHO) recently launched the global technical strategy (GTS) for malaria, which aims to reduce the incidence and mortality rates of malaria at least by 90% by 2030 [9]. Reducing the burden of malaria particularly in sub-Saharan Africa is linked to several of the sustainable development goals (SDG) [10]. To achieve the GTS and SDG malaria targets, malaria-endemic countries should have robust surveillance and health management information systems to monitor mortality and incidence rates of malaria [9]. However, Ethiopia, like many of the sub-Saharan African countries, does not strong surveillance and health management information systems to accurately measure mortality and incidence rates of malaria. In this study, the 2015 global burden of diseases, injuries and risk factors (GBD) data [11–14] were used to measure the incidence, prevalence, mortality, and disability-adjusted life years lost (DALY) rates of malaria during 1990–2015. The study has provided evidence of the performance by Ethiopia on the three MDG diseases and it can serve as a benchmark to track future progress during the SDG era.

## Methods

Ethiopia, with a population of nearly 100 million, is the second most populous country in Africa with diverse population mix and unique cultural heritage [1]. Nearly 60% of the Ethiopian population lives in malarious areas and 68% of the country’s landmass is favourable to malaria transmission [15, 16]. Malaria transmission in Ethiopia is seasonal and unstable and peaks of malaria incidence follow the major rainfall season, from July to September [15, 16].

### Data sources

The GBD 2015 utilizes comprehensive sources of data and rigorous analysis to estimate trends of cause-specific mortality rates and risk factors for 188 countries [13]. The key sources of data to model the burden of malaria in Ethiopia included verbal autopsy (VA) from the health and demographic surveillance sites (HDSS), Ethiopian Demographic and Health Surveys (EDHS), other surveys such as malaria indicator surveys (MIS) of Ethiopia and Ministry of Health reports submitted to UN agencies and published scientific articles [13].

### Causes of death modelling

Causes of death by age groups, gender and year for malaria were measured using ensemble modelling (CODEm). A detailed description of CODEm is reported elsewhere [11, 13, 17, 18]. In brief, CODEm tests a wide range of models, such as mixed effects linear models and spatial–temporal Gaussian process regression (ST-GPR) models and constructs an ensemble model based on the performance of the different models [13]. Out-of-sample predictive validity test was used to select the ensemble model for estimation of mortality rate [13]. In this model, uncertainty intervals (UI) are generated by sampling the posterior distribution of each component model in proportional to the weight of each model in the ensemble. Vital registration and VA data were corrected for garbage codes based on the GBD algorithm [13].

DALY, due to malaria, was measured by summing years of life lost (YLL) due to premature mortality and years lived with disability (YLD), a measure of non-fatal health loss, in a single metric. One DALY can be thought of as one lost year of healthy life. YLL were estimated using standard GBD methods whereby each death is multiplied by the normative standard life expectancy at each age. YLD were estimated using sequelae prevalence and disability weights derived from population-based surveys of the general public to assign disability weights to each sequela and combination of sequelae [19, 20].

## Results

The number of new cases of malaria declined from 2.8 million (95% UI 1.4–4.5 million) in 1990 to 621,345 (95% UI 462,230–797,442) in 2015. Age-standardized incidence rate among all ages and gender declined by 88.7% over the 25 years with an annualized rate of change (ARC) of 8.7%. Malaria caused an estimated 30,323.9 deaths (95% UI 11,533.3–61,215.3) in 1990 and 1561.7 deaths (95% UI 752.8–2660.5) in 2015, a 94.8% reduction over the 25 years. Age-standardized mortality rate of malaria has declined by 96.5% between 1990 and 2015 with an ARC of 13.4% (Table 1).

**Table 1:** Mortality, incidence, DALY and prevalence rates of malaria and annualized rates of change between 1990 and 2015

Measure	1990 # or rate (95% UI)			2015 # or rate (95% UI)			% change between 1990 and 2015	ARC (95% UI)		
# deaths	30,323	11,533	61,215	1561	752	2690	−94.85			
Age-standardized mortality rate/100,000	56.20	24.84	107.79	1.98	0.94	3.65	−96.47	−13.38%	−0.165	−0.101
Incidence cases	2,780,765.95	1,405,414.30	4,515,605.76	621,345.20	462,230.28	797,442.65	−77.66			
Age-standardized incidence rate/100,000	4486.69	2279.78	7045.74	504.07	372.61	637.02	−88.77	−8.75%	−0.106	−0.068
#DALY	2,223,549.25	758,961.05	4,685,892.11	184,456.15	121,905.11	264,397.55	−91.70			
Age-standardized DALY rate/100,000	3014.46	1244.54	5810.15	154.43	103.16	226.22	−94.88	−11.89%	−0.145	−0.087

**Fig. 1:**
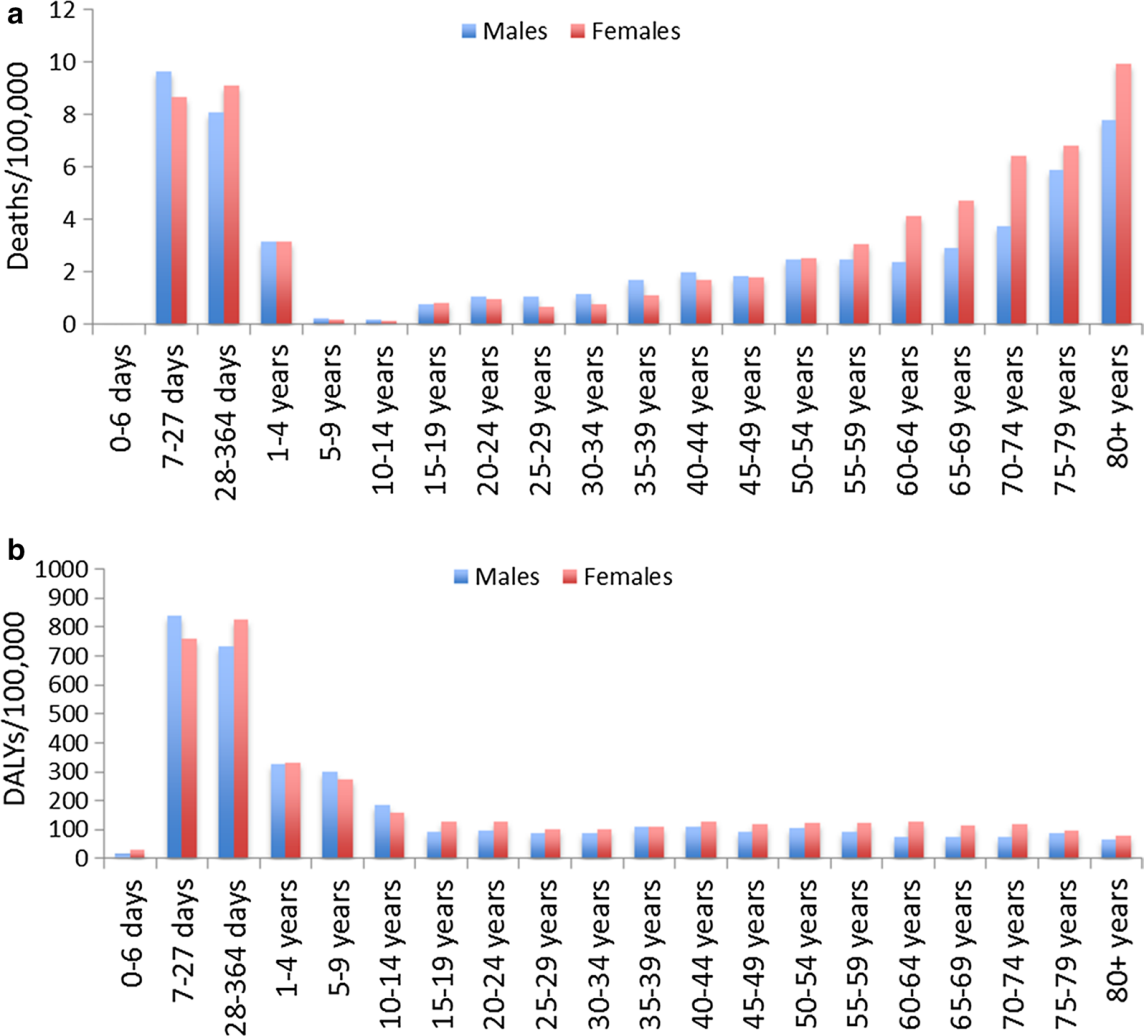
Mortality (**a**) and DALY (**b**) rates by gender and age group in 2015

**Fig. 2:**
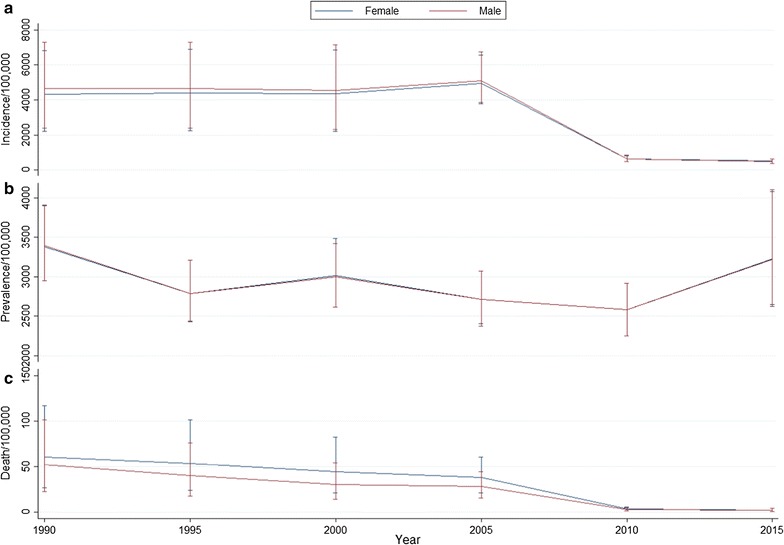
Age-standardized malaria incidence (**a**), prevalence (**b**) and mortality (**c**) rates in males and females between 1990-and 2015

Malaria mortality rate was highest among neonates (7–27 days), post-neonatal infants (28–364 days) and older individuals (≥65 years) and lowest among individuals 10–14 years in both gender (Fig. 1). The reduction of age-standardized incidence and mortality rates of malaria were more marked between 2005 and 2010. Unlike mortality and incidence rates, little reduction (5%) was observed for the age-standardized prevalence rate over the last 25 years (Fig. 2).

**Fig. 3:**
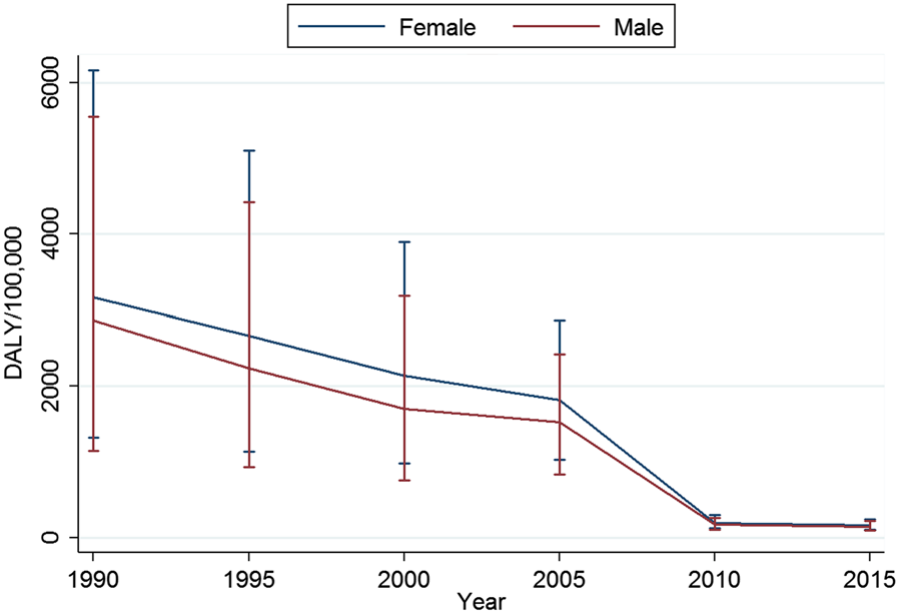
Age-standardized DALY rates of malaria by gender and year

The number of DALY due to malaria decreased from 2.2 million (95% UI 0.76–4.7 million) in 1990 to 0.18 million (95% UI 0.12–0.26 million) in 2015 with a total reduction of 91.7%. Similarly, age-standardized DALY rate declined by 94.8% during the same period (Table 1). The reduction of age-standardized DALY rate was marked during 2005 and 2010 (Fig. 3). The age-standardized DALY rate was higher among neonatal and post-neonatal period compared to the other age groups (Fig. 1).

## Discussion

The MDG targets of halving mortality rate from malaria by 2015 and efforts to reverse the incidence of this disease have been encouraging globally although there were variations among regions and countries [10]. Ethiopia has shown remarkable progress in reversing the burden and epidemics of malaria in the last two decades. Mortality and incidence rates of malaria declined by 96 and 89%, respectively, between 1990 and 2015.

Other reports also show that Ethiopia has achieved the MDG targets of malaria [1, 6, 21]. The WHO report showed a 50–75% decline in incidence and mortality rates of malaria between 2000 and 2013 [6, 21]. Between 2010 and 2015, malaria incidence and mortality rates, particularly due to *Plasmodium falciparum*, have declined by more than 50% in Ethiopia [21]. However, Ethiopia still accounts for 6% of malaria cases globally and about 12% of the global cases and deaths due to *Plasmodium vivax* occurs in Ethiopia [21]. More than 75% of deaths and cases of *P. vivax* occur in four countries: Ethiopia, Indonesia, India, and Pakistan [21].

The performance of Ethiopia in reducing the burden of malaria and reversing malaria epidemics is better than many sub-Saharan African countries [13]. Several factors could have helped Ethiopia to achieve the MDG targets. Strong government leadership in designing and implementing primary healthcare could have helped [22]. The country has implemented an innovative community-based health service delivery called health extension programme (HEP) since 2003 [22]. The HEP has trained and salaried female healthcare workers who provide basic primary healthcare services at community level. The HEP uses a Family Folder, which is a low cost and high impact health management information system at *Kebele* (lowest administrative unit) level to monitor the health service delivery and health status of the population. It contains basic household characteristics such as availability of clean drinking water, sanitation, and bed nets to prevent malaria [23]. The HEP and the Family Folder have been instrumental in making health services accessible to the poor [22–24]. The marked decline of incidence and mortality rates due to malaria since 2005 could be the effect of the HEP.

On the other hand, the significant decline of malaria incidence and mortality rates could be attributable to the effective implementation of the malaria control strategies at grassroots level. Aregawi et al. shows that malaria cases and deaths in Ethiopia substantially declined after the introduction of ACT and ITNs [4]. However, the malaria control strategy also faces several challenges to achieve WHO’s GTS targets to reduce malaria mortality and incidence rates by at least 90% by 2030 [9]. Some evidence showed that malaria transmission and incidence rate were higher in communities living around hydro-electric dams and irrigation areas in Ethiopia [25–27]. The higher prevalence of malaria in 2015 compared to that of the previous years could be the result of high malaria burden in high-risk geographic areas. This might be a challenge for a country that has a development strategy of building irrigation systems and mega-dams [28]. Although evidence shows that malaria mosquitoes are resistant to common insecticides that are used to treat bed nets [29], ITNs are still the main malaria control strategy. Hence, it is timely to consider other innovative approaches and tools to control malaria in Ethiopia. Poor community perception and awareness is also one of the main barriers to control and prevent malaria in Ethiopia which requires effective behavioural change interventions [30].

This study is based on the GBD 2015 which uses comprehensive data sources and rigorous analysis. However, the study has some limitations. First, the use of VA data in mortality estimation may introduce misclassification bias. For instance, VA could over-diagnose malaria cases [31] and exaggerate malaria deaths [32, 33]. Use of published articles could introduce publication bias since unfavorable findings may not be published.

## Conclusions

Ethiopia has achieved MDG targets related to malaria. Malaria control and treatment strategies should be intensified during the SDG-era focusing on high-risk groups and geographic areas.

## Abbreviations

ACT: artemisinin-combination therapy
ARC: annualized rates of change
CODEm: causes of death ensemble modelling
DALY: disability-adjusted life years lost
EDHS: Ethiopian Demographic and Health Survey
GBD: global burden of diseases
GTS: global technical strategy for malaria
HDSS: health and demographic surveillance system
HEP: health extension programmes
HEW: health extension workers
IPT: intermittent preventive therapy
IRS: indoor residual spray
ITN: insecticide-treated bed nets
MDG: millennium development goals
SDG: sustainable development goals
VA: verbal autopsy
WHO: World Health Organization
YLD: years of lived with disability
YLL: years of life lost due to premature mortality
